# Detritivore conversion of litter into faeces accelerates organic matter turnover

**DOI:** 10.1038/s42003-020-01392-4

**Published:** 2020-11-11

**Authors:** François-Xavier Joly, Sylvain Coq, Mathieu Coulis, Jean-François David, Stephan Hättenschwiler, Carsten W. Mueller, Isabel Prater, Jens-Arne Subke

**Affiliations:** 1 Biological and Environmental Sciences, University of Stirling, Stirling, FK9 4LA UK; 2 CEFE, Univ Montpellier, CNRS, EPHE, IRD, Univ Paul-Valéry Montpellier 3, Montpellier, France; 3 CIRAD, UPR GECO, 97285 Le Lamentin, Martinique France; 4 Chair of Soil Science, Technical University of Munich (TUM), Emil-Ramann-Str. 2, 85354 Freising, Germany; 5 Department of Geosciences and Natural Resource Management, University of Copenhagen, Øster Voldgade 10, 1350 Copenhagen K, Denmark

**Keywords:** Carbon cycle, Ecosystem ecology

## Abstract

Litter-feeding soil animals are notoriously neglected in conceptual and mechanistic biogeochemical models. Yet, they may be a dominant factor in decomposition by converting large amounts of plant litter into faeces. Here, we assess how the chemical and physical changes occurring when litter is converted into faeces alter their fate during further decomposition with an experimental test including 36 combinations of phylogenetically distant detritivores and leaf litter of contrasting physicochemical characteristics. We show that, across litter and detritivore species, litter conversion into detritivore faeces enhanced organic matter lability and thereby accelerated carbon cycling. Notably, the positive conversion effect on faeces quality and decomposition increased with decreasing quality and decomposition of intact litter. This general pattern was consistent across detritivores as different as snails and woodlice, and reduced differences in quality and decomposition amongst litter species. Our data show that litter conversion into detritivore faeces has far-reaching consequences for the understanding and modelling of the terrestrial carbon cycle.

## Abstract (web summary)

By performing an ecologically relevant experiment with variety in litter composition and detritivore species, Joly et al. find that litter conversion into faeces has a profound effect on organic matter lability and decomposition. These findings have implications for biogeochemical models of carbon cycling.

## Introduction

Plant litter decomposition is a fundamental biogeochemical process in terrestrial ecosystems^1^, occurring through three dominant pathways: leaching of water-soluble compounds, enzymatic degradation by microorganisms, and litter processing by soil animals^2^. While the leaching and microbial pathways received considerable attention^3,4^, the understanding of the soil animal pathway lags behind^5^. This litter processing is mostly driven by detritivores, i.e. soil animals that feed on decomposing litter, assimilate a part of it and typically return the largest part to the soil as faeces (‘litter conversion into detritivore faeces’ hereafter)^6^. Studies from temperate^7^, Mediterranean^8^, tropical^9^ and arid^10^ ecosystems reported that large portions of annual litterfall are consumed by detritivores and converted into faeces. This conversion entails physical changes with a reduction of initially intact litter to minute particles that constitute the faeces (referred to as comminution or fragmentation in the literature^2^) and chemical changes with a partial digestion of the litter passing through detritivore guts^6^. Because litter physicochemical characteristics (‘quality’ hereafter) predominantly control the leaching and microbial pathways, quality changes driven by detritivores may profoundly alter the further fate of the converted litter. Yet, the consequences of this conversion for decomposition processes are poorly understood and the few studies addressing this question reported conflicting results^6,11,12^. The lack of consensus may be attributed to two unresolved questions: Does initial litter quality determine the further decomposition of detritivore faeces after conversion? Does the conversion effect on faeces decomposition vary among detritivore species? With these two questions presently unanswered, the potentially fundamental role of detritivores in biogeochemical cycling and soil carbon dynamics remains elusive and difficult to predict.

A recent study evaluating how an abundant and widespread millipede species modifies decomposition reported accelerated rates of carbon (C) and nitrogen (N) loss from faeces compared to intact litter from seven tree species^11^. Most importantly, the effect of litter conversion into detritivore faeces on further decomposition was particularly strong for recalcitrant litter. This has far-reaching implications as it suggests that the dominant litter quality control over decomposition^13,14^ may be strongly altered following litter conversion into faeces. This is of critical importance given that litter quality control over decomposition occupies a key position in conceptual and mechanistic decomposition models^15^. However, the focus of the study on a single animal species prevents generalising this pattern.

Although it is currently assumed that detritivore processing of litter, thus, including the conversion effect, is similar among detritivore species^2^, it seems reasonable to assume that the large interspecific differences in anatomy, assimilation^6^ and faeces characteristics^6,16^ observed among different species of detritivores may influence the effect that litter conversion into detritivore faeces will have on further decomposition. Indeed, the astounding diversity of soil organisms is increasingly recognised as an important variable in determining ecosystem processes^17^. Yet, how the conversion effect varies among detritivores and how this detritivore identity control interacts with litter quality is unknown.

**Fig. 1:**
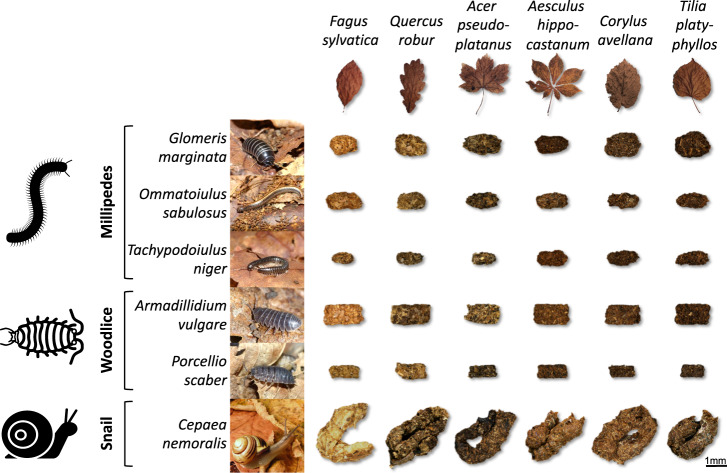
Faeces diversity as a function of litter and detritivore identity. Diversity of leaf litter (from six tree species) and detritivores (three millipede (Diplopoda), two woodlouse (Crustacea) and one snail (Gastropoda) species) included in this study, with the respective faeces from all possible combinations of litter and detritivore species. Faeces are to scale; animals and leaves are not to scale.

Here we address this knowledge gap by feeding six phylogenetically and physiologically different detritivore species (to maximise potential functional differences among species) with six different litter types (to form a litter quality gradient), collecting the resulting 36 faeces types (Fig. 1) and comparing their quality (11 physicochemical characteristics) and decomposition rates (C and N losses) with that of the six intact litter types. We hypothesised that (H1) litter conversion into detritivore faeces generally increases litter quality and consequently C and N losses and that (H2) this increase is stronger for recalcitrant and slow-decomposing litter, regardless of detritivore species.

## Results

### Changes in quality

**Fig. 2:**
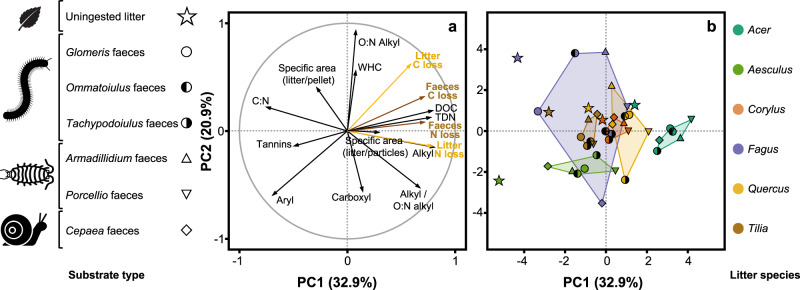
Physicochemical characteristics of faeces and intact litter. Principal component analysis (PCA) of physicochemical characteristics for all substrates (6 intact litter and 36 faeces treatments, Fig. 1). **a** displays the variable loadings (dark-grey arrows) and the correlations of intact litter and faeces C and N losses with the PCA axes (yellow and brown arrows for intact litter and faeces, respectively). **b** displays the scores for all substrates (mean; *n* = 3), with coloured convex hulls containing, for each litter species, faeces types derived from all detritivore species; intact litter is displayed separately. WHC water-holding capacity, DOC dissolved organic carbon, TDN total dissolved nitrogen. Variables were centred and standardised prior to ordination.

**Fig. 3:**
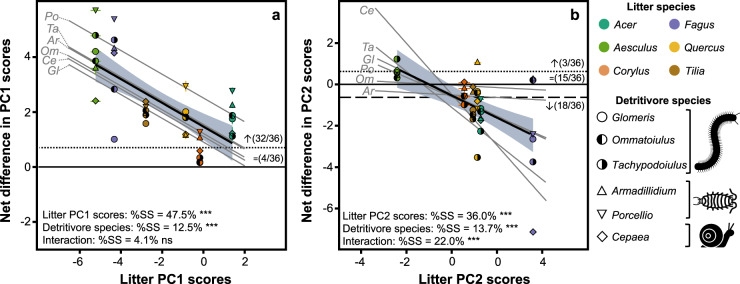
Net effect of litter conversion into faeces on physicochemical characteristics. Net differences in principal component (PC) scores (see Fig. 2) between faeces and the leaf litter from which they were derived as a function of litter PC1 scores (**a**) and PC2 scores (**b**) for all combinations of litter and detritivore species (mean ± SE; *n* = 3). Differences above the black dotted lines are significantly positive, and differences below the black dashed line are significantly negative. Numbers in brackets indicate the relative frequency of non-significant differences (=), positive differences (↑) and negative differences (↓). %SS (percentage of total sum of squares) and asterisks (ns: *P* > 0.05; ***P < 0.001) indicate the variance and *P* value associated with the effect of litter quality (PC scores), detritivore species and their interaction in a two-way ANCOVA. Thick black lines represent the regression lines for all treatments, with grey areas representing the 95% confidence intervals of regression lines. Grey lines represent the regression line for each detritivore species separately, each labelled with the first two letters of the detritivore genus name.

The 36 faeces types varied in size, shape, colour (Fig. 1) and quality (Fig. 2) depending on litter and detritivore species. Visually, the faeces colour was clearly determined by the litter species, independent of the detritivore species from which they were derived, with light colour when detritivores were feeding on *Fagus* litter and dark colour when feeding on *Tilia* litter (Fig. 1). In contrast, the shape and size of the faeces varied with detritivore species, with ovoids (length ca. 0.8–1.0 mm) for millipedes, rectangular cuboids (length ca. 0.8–1.3 mm) for woodlice and cylinders (length ca. 2.8 mm) for the snail (Fig. 1). The principal component analysis (PCA) of litter and faeces characteristics (Fig. 2a and see Supplementary Table 1 for detailed summary of characteristics), including elemental composition (C/N ratio, concentrations of dissolved organic carbon (DOC), total dissolved nitrogen (TDN)), chemical properties (tannin concentration, five chemical shift regions of ^13^C nuclear magnetic resonance (^13^C-NMR) spectra) and physical characteristics (specific area, water-holding capacity (WHC)), revealed that the 42 substrates (6 intact litter controls and 36 faeces types) predominantly differed along the first principal component (PC1) in their elemental composition. This PC1, which captured 32.9% of the total variability, represented the range in C/N ratio (17.6–42.8), tannin concentrations (2.1–24.7 mg/g), DOC concentrations (3.5–26.4 mg/g) and TDN concentrations (0.5–4.9 mg/g) and separated substrates with high C/N ratio and tannin concentrations on the negative end from substrates with high DOC and TDN concentrations on the positive end (Fig. 2a). On this PC1, scores were significantly higher (*P* < 0.001; Student’s *t* test) for faeces (0.3 on average) than for intact litter (−2.0 on average; Fig. 2b). Scores for intact litter differed significantly (*P* < 0.001; one-way analysis of variance (ANOVA)) among litter species (ranging from −5.2 for *Aesculus* litter to 1.4 for *Acer* litter). Faeces scores also differed significantly (*P* < 0.001; one-way ANOVA) among faeces types (ranging from −3.3 for *Glomeris* faeces derived from *Fagus* litter to 4.2 for *Porcellio* faeces derived from *Acer* litter). The net difference in PC1 scores between faeces and intact litter from which the faeces were derived was significantly positive for 32 of the 36 faeces types (Fig. 3a). This difference was negatively related with litter PC1 scores, which explained 47.5% of the variance (Fig. 3a; analysis of covariance (ANCOVA)), resulting in large positive differences for faeces derived from litter with low PC1 scores (e.g. *Aesculus*) and smaller or even non-significant differences for faeces derived from litter with high PC1 scores (e.g. *Acer*; Fig. 3a). This relation was particularly steep for the millipede *Tachypodoiulus* and the snail *Cepaea* (significant interaction with detritivore species; Fig. 3a; ANCOVA), and differences also varied in magnitude among detritivore species (Fig. 3a; ANCOVA).

Along the second principal component (PC2), which captured 20.9% of the total variability, substrates differed in their physical and chemical properties. Specifically, this PC2 represented the range in WHC (1.53–3.26 g H_2_O/g), O/N alkyl C (43.8–68.1% of total C; representing the polysaccharide content) and carboxyl C (2.0–16.3% of total C; representing the content of oxidised C) and separated substrates with high WHC and O:N alkyl content on the positive end from substrates with high carboxyl content on the negative end (Fig. 2a). Scores were significantly lower (*P* < 0.05; Student’s *t* test) for faeces (−0.1 on average) than for intact litter (0.9 on average, Fig. 2b). Scores for intact litter differed significantly (*P* < 0.001; one-way ANOVA) among litter species (ranging from −2.4 for *Aesculus* litter to 3.6 for *Fagus* litter). Faeces scores also differed significantly (*P* < 0.001; one-way ANOVA) among faeces types (ranging from −3.6 for *Cepaea* faeces derived from *Fagus* litter to 3.8 for *Ommatoiulus* faeces derived from *Fagus* litter). The net difference in PC2 scores between faeces and intact litter from which the faeces were derived was significantly positive for 3 of the 36 faeces types, not different from zero for 15 faeces types and significantly negative for the remaining 18 faeces types. Similar to what we observed for PC1 scores, the difference between faeces and intact litter was strongly and negatively related with litter PC2 scores, which explained 36.0% of the variance (Fig. 3b; ANCOVA), resulting in positive differences for faeces derived from litter with low PC2 scores (e.g. *Aesculus*) and large negative differences for faeces derived from litter with high PC2 scores (e.g. *Fagus*; Fig. 3b). This relation was particularly steep for the millipede *Tachypodoiulus* and the snail *Cepaea* (significant interaction with detritivore species, Fig. 3b; ANCOVA), and differences varied in magnitude among detritivore species (Fig. 3b; ANCOVA).

### Changes in carbon and nitrogen dynamics during decomposition

**Fig. 4:**
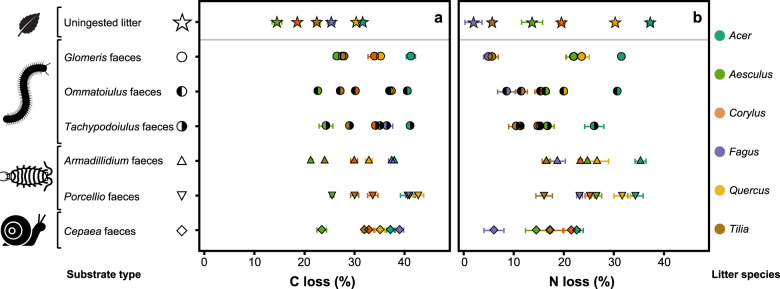
Decomposition of faeces and intact litter. Carbon (**a**) and nitrogen (**b**) losses for all substrates (6 intact litter and 36 faeces types; see Fig. 1) (mean ± SE; *n* = 5). Non-visible error bars are smaller than the symbol.

**Fig. 5:**
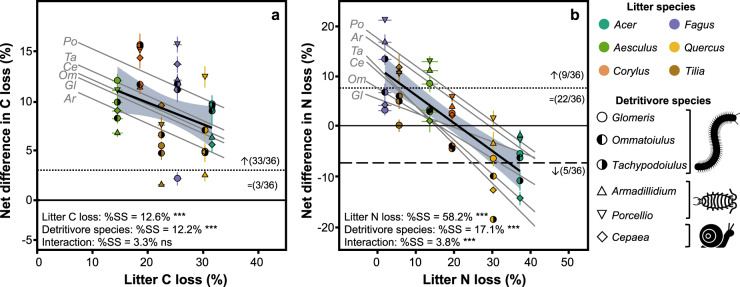
Net effect of litter conversion into faeces on decomposition. Net differences in **a** C loss and **b** N loss (see Fig. 4) between faeces and the intact litter from which faeces are derived as a function of litter C and N loss, respectively, for all faeces types (mean ± SE; *n* = 5). Differences above the black dotted lines are significantly positive, and differences below the black dashed line are significantly negative. Numbers in brackets indicate the relative frequency of non-significant differences (=), positive differences (↑) and negative differences (↓). %SS (percentage of total sum of squares) and asterisks (ns: *P* > 0.05; ***P < 0.001) indicate the variance and *P* value associated with the effect of litter C or N loss, detritivore species and their interaction in a two-way ANCOVA. Thick black lines represent the regression lines for all treatments, with grey areas representing the 95% confidence intervals of regression lines. Grey lines represent the regression line for each detritivore species separately, each labelled with the first two letters of the detritivore genus name.

After 180 days of incubation under controlled conditions, faeces lost significantly more of their initial C than intact litter (*P* < 0.001; Student’s *t* test), with an average C loss of 32.9% for faeces compared to 23.8% for intact litter across treatments, representing a 38.1% increase in C loss following litter conversion into faeces (Fig. 4a). Litter C loss differed significantly among litter species (*P* < 0.001; one-way ANOVA), ranging from 14.5% of initial C lost for *Aesculus* litter to 31.6% for *Acer* litter. Faeces C loss also differed significantly among faeces types (*P* < 0.001; one-way ANOVA), ranging from 21.2% of initial C loss for *Armadillidium* faeces derived from *Aesculus* litter to 42.7% for *Porcellio* faeces derived from *Quercus* litter. Litter C loss was positively related with both PCA axes 1 and 2, which explained 34.1% (*P* < 0.001) and 39.0% (*P* < 0.001) of the variance, respectively (Fig. 2a). Faeces C loss was also positively related with both PCA axes but more so with the first axis explaining 53.0% (*P* < 0.001; linear regression) of the variance, compared to 10.8% (*P* < 0.001; linear regression) for the second axis (Fig. 2a). Notably, faeces C loss was tightly related to DOC concentration (Fig. 2a). The net difference in C loss between faeces and the intact litter from which the faeces derived was significantly positive for 33 of the 36 faeces types (not different from zero for the remaining three types, Fig. 5a). This difference was negatively related with litter C loss, which explained 12.6% of the variance (Fig. 5a; ANCOVA), resulting in large positive differences for faeces derived from litter with low C loss (e.g. *Aesculus*) and smaller or even non-significant differences for faeces derived from litter with high C loss (e.g. *Acer*; Fig. 5a). This relation was similar for all detritivore species (no interaction with detritivore species; Fig. 5a; ANCOVA) but varied in magnitude among detritivore species (Fig. 5a; ANCOVA).

In contrast to C loss, N loss did not differ significantly between faeces and intact litter across treatments (*P* = 0.377; Student’s *t* test), with an average N loss of 19.7% for faeces compared to 18.1% for intact litter. Litter N loss differed significantly among litter species (*P* < 0.001; one-way ANOVA), ranging from 2.0% of initial N lost for *Fagus* litter to 37.3% for *Acer* litter (Fig. 4b). Faeces N loss also differed significantly among faeces types (*P* < 0.001; one-way ANOVA), ranging from 5.0% of initial N loss for *Glomeris* faeces derived from *Fagus* litter to 35.3% for *Armadillidium* faeces derived from *Acer* litter (Fig. 4b). Litter and faeces N losses were both positively related with the first PCA axis, which explained 60.2% (*P* < 0.001; linear regression) and 52.7% (*P* < 0.001; linear regression) of the variance, respectively (Fig. 2a). Even without different N losses from intact litter and faeces on average across treatments, the change in N loss showed notable variation across the 36 faeces types, with significant increases in N loss compared to intact litter for 9, significant decreases for 5 and no differences for 22 faeces types. Similar to C loss, the difference in N loss between faeces and intact litter was strongly and negatively related with litter N loss, which explained 58.2% of the variance (Fig. 5b; ANCOVA), resulting in large positive differences for faeces derived from litter with low N loss (e.g. *Fagus*) and large negative differences for faeces derived from litter with high N loss (e.g. *Acer*; Fig. 5b). In contrast to C loss, this relation varied with detritivore species (significant interaction with detritivore species, Fig. 3b; ANCOVA) and was particularly steep for the millipede *Tachypodoiulus* and the snail *Cepaea*. Differences in N loss between faeces and intact litter also varied in magnitude among detritivore species (Fig. 3b; ANCOVA).

## Discussion

Our large and representative experiment with 36 different types of detritivore faeces revealed clear patterns of C and N loss in response to litter conversion into faeces. Specifically, we showed that C cycling was accelerated by 38.1% on average over a 6-month decomposition period when detritivores converted leaf litter into faeces, in line with our first hypothesis. This result provides a clear answer to a long-standing debate about the consequences of litter conversion into faeces for further decomposition: Does the conversion accelerate decomposition by increasing the surface area available to microbial colonisation^18,19^ or does it decelerate decomposition by depleting readily degradable compounds^6,12^? To date, the limited and contrasting data on this effect made this debate largely unresolved. The 7 published studies with a total of 16 combinations of litter and detritivore species reported slower, faster or unchanged decomposition in faeces compared to intact litter for 3^20,21^, 9^11,21–23^, and 4^11,23–25^ of these combinations, respectively. Here, with significantly faster C cycling in faeces compared to intact litter for 33 litter-detritivore combinations and unchanged C cycling for the other 3, our study nearly triples the existing data and suggest that unchanged or slower decomposition is more the exception than the rule.

The concomitant assessment of changes in a wide range of physicochemical characteristics following litter conversion into faeces provides an unprecedented insight into the potential mechanisms driving this accelerated decomposition. This conversion effect, which comprises the effects of litter comminution, partial digestion and repackaging into faeces, significantly increased the organic matter lability (reduced C:N ratio and tannin concentrations and increased DOC and TDN concentrations; Figs. 2 and 3) in all but 4 of the 36 faeces types, as expected by our first hypothesis. Moreover, carbon loss from faeces appeared to be mostly related to this lability (Fig. 2a). Interestingly, while this lability axis represented an increase in surface area resulting from the comminution of leaves into minute particles, it mostly represented an increase in DOC concentration, which was the variable most tightly correlated with faeces C loss. This suggests that litter conversion into detritivore faeces may accelerate decomposition mostly by facilitating the leaching pathway rather than the microbial pathway, as commonly assumed. This is noteworthy, as the lack of evidence for a higher microbial activity in faeces compared to intact litter^20,26–28^ was one of the main reasons recent reviews argued against the assumption that litter conversion into faeces accelerates decomposition^6,12^. Here we present compelling evidence that litter conversion into detritivore faeces does accelerate decomposition and propose that increased leaching may be one of the dominant underlying mechanisms.

In light of the emerging paradigm of soil organic matter (SOM) formation, which posits that decomposition products such as leachates and microbial products, not litter recalcitrance, drive the formation and stabilisation of SOM^29^, our results suggest that detritivores may stimulate SOM formation. Indeed, by increasing leaching and microbial degradation, litter conversion into detritivore faeces could be a springboard for further production of decomposition products thereby facilitating the formation of stable SOM. In addition, other mechanisms associated with litter conversion into faeces, which we did not study here explicitly, may affect the fate of detritivore faeces and their role for SOM dynamics. These include the mixing of organic matter and minerals in faeces due to detritivores ingesting mineral soil particles along with plant litter; the burial of faeces (active or passive), which may improve conditions for further decomposition^23,30^; the production of leftover litter parts (e.g. leaf veins) for which exposure to leaching and microbial degradation may be increased and the further ingestion of faeces by other soil animals^26^. In turn, our experiment included the removal of leaf veins from the intact litter, which may have partially fragmented the decomposing material and falsely inflated the intact litter decomposition thereby underestimating the litter-to-faeces conversion effect. It is also important to note that for this experiment we employed litter species collected at the same time as the detritivore species for maximised realism. A drawback of this choice is that the different litter species varied somewhat in decomposition stages. This could have led to converging initial quality of intact litter and a potentially underestimated conversion effect. Moreover, it is currently unknown how the conversion effect varies through time and progress in the decomposition process. Last, a variable part of the ingested litter is assimilated by detritivores and later respired or incorporated into animal biomass. A more thorough quantification of the assimilated part of litter and its fate will be necessary to evaluate the overall effect of detritivores on decomposition and SOM formation^6,31^. All these mechanisms and methodological aspects require further investigation for an integrated understanding of the effect of detritivores on decomposition and SOM formation.

A key result of our study, the first of its kind to include both litter and detritivore diversity, is that the direction and magnitude of the effect of litter conversion into detritivore faeces on quality and decomposition can largely be predicted by the characteristics of the litter being consumed. Specifically, we found that the increase in lability (decrease in C:N ratio and tannin concentration and increase in leachate concentrations) and carbon loss following litter conversion into detritivore faeces was stronger for recalcitrant and slow-decomposing litter, in line with our second hypothesis, and null for more labile and fast-decomposing litter. Similar patterns were previously reported for microbial activity in faeces^32^ and their quality and decomposition^11^ using a single detritivore species. Here we extend these previous findings by showing strikingly similar effects across a range of detritivore species that vary in many aspects, including their phylogenetic position, physiology and ecology. The important role that initial litter properties have for the expression of the litter-to-faeces conversion effect may explain the discrepancies between previous studies that found effects ranging from positive to negative, often based on one single litter type. Actually, the pattern we report does not exclude the possibility of even negative effects of litter conversion into faeces if the litter quality gradient were pushed even further to more labile and fast-decomposing litter. In fact, the few negative effects on decomposition reported previously were for freshly senesced *Alnus glutinosa* and *Salix caprea* litter^20,21^, which are nutrient rich and rapidly decomposing. On the other end of the spectrum, positive effects reported in other studies were observed for more recalcitrant litter^11,21–23^. Although these studies differed in other aspects, litter dependency appears to be key in reconciling the contrasting results of past studies. This litter dependency was even clearer for quality along the second PCA axis and for N dynamics. Particularly, N was lost at lower rates in faeces compared to intact litter for fast-decomposing litter, while slow-decomposing litter produced faeces that lost N at higher rates. Consequently, across all litter species, the overall average N loss was not different before and after litter conversion into faeces, opposite to our first hypothesis. The fact that litter conversion into detritivore faeces increased N loss and quality for recalcitrant and slow-decomposing litter and decreased N loss and quality for more readily degradable litter suggests that detritivores equilibrate differences in N release among litter types. A potential mechanism underlying this litter-dependency may be that the comminution of leaf litter into minute particles removes physical protection^33^, thereby increasing leaching and consequently decomposition. Because leaching is typically lower for recalcitrant and slow-decomposing litter due to such physical protection^34^, litter comminution may have a particularly strong effect on the decomposition of recalcitrant and slow-decomposing litter. Collectively, our results indicate that litter conversion into detritivore faeces increases quality and decomposition for recalcitrant and slow-decomposing litter, while it does not change or even reduce quality and decomposition for labile and fast-decomposing litter, thereby reducing differences in quality and decomposition among litter species. An important consequence of this homogenisation is that, in ecosystems with abundant detritivore communities, the control of litter quality on decomposition—a key parameter in global decomposition models^15^—is substantially attenuated.

Detritivores constitute a very large group of soil animals of high taxonomic diversity. The role of this diversity and the differences among individual species in the process of litter conversion into faeces and its consequences for decomposition is poorly documented. Here we show that homogenisation is consistent across detritivore species, both within and across phylogenetic groups, at least for our six studied species, which are common in European temperate and Mediterranean ecosystems. Nevertheless, the magnitude of this effect on litter quality (Fig. 3) and decomposition rates (C and N losses, Fig. 5) varied to some extent among the studied detritivore species, with detritivore identity explaining 12–17% of the variance in the change in quality and decomposition. Although our design did not allow statistical testing for differences among the phylogenetic groups (Gastropoda, Crustacea, Diplopoda) included in our test, we noted that the change in N loss was particularly high for the woodlouse species (Crustacea; Figs. 4b and 5b). Additionally, for N cycling and the second PCA axis, the strength of the homogenisation on quality and decomposition varied to some extent among detritivore species, as shown by the interaction between detritivore identity and litter parameter accounting for 4–22% of the variance in the change in quality and decomposition. This indicates that detritivore identity may modulate the strong general homogenisation to some extent. Identifying detritivore characteristics that underpin these differences (e.g. consumption rate, assimilation efficiency, gut microbiome, faeces characteristics) thus appears as an important perspective for future work to predict the contribution of detritivores to decomposition, thereby contributing to the ongoing effort of defining terrestrial invertebrates’ functional characteristics that affect ecosystem functioning^35,36^. This may permit the approach of character-matching^37^, in which combining the characteristics of detritivores with those of the litter they feed on would allow integrating the effect of detritivore identity and its interaction with litter quality into the modelling of litter decomposition. Collectively, given the prevalence of litter conversion into detritivore faeces in many terrestrial ecosystems and the magnitude of the associated changes in quality and decomposition that depend on litter and detritivore identity, we argue that the explicit inclusion of litter processing by detritivores and associated homogenisation in decomposition and SOM models is key to improving their predictive capabilities.

## Methods

### Detritivore and leaf litter collection

We collected six phylogenetically diverse species of detritivores in various areas of the Scottish Lowlands in May and June 2018, including three millipede species (Diplopoda), two woodlouse species (Crustacea) and one snail species (Gastropoda). Millipede species include the common pill millipede (*Glomeris marginata* (Villers, 1789)) collected near Peebles, UK (55°38′45.8″N, 3°07′55.4″W), the striped millipede (*Ommatoiulus sabulosus* (Linnaeus, 1758)) collected near Dunfermline, UK (56°02′23.7″N, 3°19′49.2″W) and the white-legged millipede (*Tachypodoiulus niger* (Leach, 1815)) collected near Dundee, UK (56°32′08.5″N, 3°01′51.9″W). Woodlouse species include the common pill woodlouse (*Armadillidium vulgare* (Latreille, 1804)) collected near Dunfermline, UK (56°01′35.3″N 3°23′14.1″W) and the common rough woodlouse (*Porcellio scaber* (Latreille, 1804) collected in Stirling, UK (56°07′26.7″N, 3°55′51.2″W). The snail species was the brown-lipped snail (*Cepaea nemoralis* (Linnaeus, 1758)) collected in Stirling, UK (56°08′07.3″N, 3°55′16.3″W). These species are common in diverse ecosystems across Mediterranean and temperate ecosystems in Europe, where they feed on decomposing litter and produce large amounts of faeces^16,38–40^. Detritivores were kept in plastic boxes and fed with moist litter from various tree species from their respective collection sites before the start of the experiment.

To obtain a gradient of leaf litter quality, we collected leaf litter from six deciduous broadleaf tree species in the Scottish Lowlands. These species include sycamore maple (*Acer pseudoplatanus*, L.), horse chestnut (*Aesculus hippocastanum*, L.), common hazel (*Corylus avellana*, L.), European beech (*Fagus sylvatica*, L.), English oak (*Quercus robur*, L.) from a woodland near Dundee, UK (56°32′08.5″N, 3°01′51.9″W) and lime (*Tilia platyphyllos*, L.) from a woodland in Stirling, UK (56°08′29.5″N, 3°55′14.2″W). Because detritivores are most active in spring and summer in these ecosystems, they feed on partially decomposed litter, which they prefer over freshly fallen litter (David and Gillon^8^). We thus collected leaf litter from the forest floor in May 2018, air-dried it and stored it in cardboard boxes until use.

### Faeces production

To compare the quality and decomposability of leaf litter with faeces derived from the same litter and produced by diverse detritivore species, we set up two series of boxes for the production of the needed material. In the first of these series, we placed each detritivore species together with each litter species to produce the 36 different faeces types (Fig. 1; 6 litter species × 6 detritivore species = 36 faeces types). The second of these series contained the litter species only without any detritivores to produce intact litter from each tree species (6 litter species) under the same conditions for the same amount of time. In total, 42 different substrates were generated. To do so, we placed ca. 30 g of air-dry leaf litter from each species separately in plastic boxes (30 cm × 22 cm × 5.5 cm) to which we added ca. 50 individuals from each detritivore species separately or no detritivore for the intact litter treatment. We sprayed the litter with water to optimise litter moisture for detritivore consumption while avoiding water accumulation at the bottom of the boxes. We kept the boxes at room temperature (ca. 20 °C) for 4 weeks and collected the produced faeces/intact litter twice a week. For the faeces, we placed the content of each box in a large bucket and gently agitated to let detritivores and faeces fall to the bottom of the bucket. After collecting the faeces, we placed all the leaf litter and detritivores back into their boxes and sprayed the litter with water to keep moisture conditions constant. For the intact litter treatment, we followed the same procedure but collected just three random leaves out of the buckets. After each collection step, the combination-specific pools of leaf litter and faeces were dried at 30 °C. At the end of the faeces production period, we manually removed small leaf litter fragments from all combination-specific pools of faeces. Additionally, because detritivores feed on leaf lamina and leave leaf veins mostly uneaten^6^, we cut out the veins from the species-specific pools of intact leaf litter. This was done to ensure the comparability of quality and decomposability between faeces and intact litter.

### Litter and faeces quality

To evaluate the effect of litter conversion into detritivore faeces on organic matter quality, we compared the quality of faeces to that of intact litter by measuring a series of physical and chemical quality parameters on all 42 substrates (6 litter species + 36 faeces types). Chemical characteristics included total carbon (C) and nitrogen (N) concentrations, DOC and TDN concentrations, total tannin concentrations, and ^13^C solid-state NMR spectra. Physical characteristics included WHC and specific area (surface area per unit of mass). Prior to these measurements, we drew three subsamples from each pool of substrate type. A part of each subsample was ground using a ball mill (TissueLyser II, Qiagen) to measure total C, N and tannin concentration and generate NMR spectra. The other part of each subsample was kept intact and used for all other measurements. All measurements were thus done on these three subsamples per substrate type, except for NMR spectra that were measured once per substrate type on a sample made by pooling all three ground subsamples. This pooling was necessary to obtain a sample large enough for the NMR analyses. Total C and N concentrations were measured with a flash CHN elemental analyser (Flash Smart, ThermoScientific). To measure DOC and TDN, we extracted leachates by placing ca. 30 mg of air-dried material with 25 ml of deionised water in 50 ml Falcon tubes and agitating the tubes horizontally on a reciprocal shaker for 1 h. Water extracts were then filtered through 0.45-μm cellulose nitrate filters to isolate the leachate fraction. Concentrations of DOC and TDN in leachates were measured with a TOC analyser (Shimadzu, Kyoto, Japan) equipped with a supplementary module for N. Tannin concentrations were measured with the protein-precipitable phenolics microplate assay, a microplate protocol adapted from Hagerman and Butler^41^. We obtained ^13^C-NMR spectra by applying ^13^C cross-polarisation magic angle spinning NMR spectroscopy using a 200 MHz spectrometer (Bruker, Billerica, USA). The samples were spun in 7 mm zirconium dioxide rotors at 6.8 kHz with an acquisition time of 0.01024 s. To avoid Hartmann–Hahn mismatches, a ramped ^1^H impulse was applied during a contact time of 1 ms. We applied a delay time of 2.0 s and the number of scans was set to 1500, yet some of the samples required longer measurements due to the low amount of sample material; in this case, we multiplied the number of scans to 3000, 6000 or 15000. As reference for the chemical shift, tetramethylsilane was used (0 ppm). We used the following chemical shift regions to integrate the spectra: −10–45 ppm alkyl C, 45–110 ppm O/N alkyl C, 110–160 ppm aromatic C, and 160–220 ppm carboxylic C. We measured the WHC by placing ca. 15 mg of air-dried intact material with 1.5 ml of deionised water in 2 ml Eppendorf tubes, agitating the tubes horizontally on a reciprocal shaker for 2 h, retrieving the material and placing it on a Whatman filter to remove excess water, weighing the wet material and reweighing it after drying at 65 °C for 48 h. We measured the specific area of leaf litter, faecal pellets and faeces particles from photographs using a stereomicroscope (ZEISS STEMI 508). For leaf litter and faecal pellets, we took photographs of ca. 20 mg of air-dried intact material. To visualise faeces particles, we weighed ca. 1 mg of air-dried faecal pellets and placed them in a beaker with 20 ml of deionised water for 2 h, allowing complete dissolution of the faecal pellets. We then filtered the faeces particles and photographed the filters under a stereomicroscope. Dimensions of each litter pieces and faecal pellets/faeces particles were measured using the image analysis software (ImageJ, version 1.46r). For all substrate types, we divided the calculated surface area by the dry mass of the sample to obtain the specific area.

### Faeces and litter decomposition parameters

To evaluate the effect of litter conversion into detritivore faeces on C and N cycling, we compared the C and N loss of faeces to that of intact litter by incubating all 42 substrates in microcosms under controlled conditions for 6 months (180 days). Microcosms consisted of 250-ml plastic containers filled with 90 mg of air-dry soil collected from a temperate grassland (56°8′40.1″N, 3°54′50.9″W). We chose this soil to avoid any home-field advantage effect as this soil did not receive litter input from any of the studied tree species and none of the selected soil animals were present at this site. About 120 mg of each substrate were placed separately within a small polyvinyl chloride tube (30 mm diameter × 30 mm height) closed in the bottom with a 100-µm mesh and left open on the top. Each tube was then placed on top of the soil within the microcosm. Five replicates per substrate were prepared, resulting in a total of 210 microcosms (42 substrates × 5 replicates). Microcosms were watered by adding water directly over the tube containing faeces/litter so as to reach 70% of soil WHC and incubated at 22 °C and 70% relative humidity in a controlled environment chamber. To limit desiccation while ensuring gas exchange, we drilled four 3-mm holes in each microcosm cap. These microcosms were then weighed weekly and watered to their initial weight at 70% soil WHC. We placed replicates on separated shelves according to a randomised complete block design. Both block positions within the controlled environment chamber and microcosm positions within blocks were randomised weekly. After 180 days, remaining intact litter and faeces in microcosms were collected, dried at 30 °C for 48 h, weighed and ground with a ball mill (TissueLyser II, Qiagen). We measured C and N concentrations in all samples with a flash CHN Elemental Analyser (Flash Smart, ThermoScientific). The percentage of C and N lost after the incubation was calculated as:

$$\frac{{M_{\rm{i}} \times {\rm{CN}}_{\rm{i}} - M_{\rm{f}} \times {\rm{CN}}_{\rm{f}}}}{{M_{\rm{i}} \times {\rm{CN}}_{\rm{i}}}} \times 100,$$

where *M*_i_ and *M*_f_ are the initial and final 30 °C dry masses, respectively, and CN_i_ and CN_f_ are the initial and final C or N concentrations, respectively.

### Statistics and reproducibility

To visualise how the 11 physicochemical characteristics were related and how their values differed between all substrates, we used a PCA, with all variables centred and standardised prior to ordination. Because NMR spectra were measured on a composite sample combining the three replicates of each substrate, a unique value was attributed to all replicates for each NMR region.

To test our first hypothesis, we tested the overall effect of substrate form (faeces vs. intact litter) on quality (scores on PC1 and PC2) and decomposition (C and N losses) of all substrates using Student’s *t* tests. To identify the faeces types with significantly different quality (scores on PC1 and PC2) and decomposition (C and N losses) compared to that of the intact litter from which the faeces were derived, we tested the effect of substrate identity (all 42 substrates included as individual levels) on quality (scores on PC1 and PC2) and decomposition (C and N losses) using one-way ANOVAs. We then used Tukey’s honestly significant difference tests to determine significant differences between each faeces type and the corresponding intact litter.

To test our second hypothesis, we expressed the changes in quality and decomposition following litter conversion into detritivore faeces as net differences in quality (scores on PC1 and PC2) and decomposition (C and N losses) between faeces and the litter from which faeces were derived. We then compared the hypothesised role of intact litter quality/decomposition (PC1 and PC2 scores, C and N losses) and the role of detritivore species on changes in quality/decomposition (net differences in PC1 and PC2 scores, C and N losses) by performing ANCOVAs with intact litter quality/decomposition as the continuous variable and detritivore species as categorical variable (all six detritivore species as individual levels). For all ANVOCAs, the variance associated with each term (intact litter quality/decomposition; detritivore species; interaction) was computed by dividing the sum of squares by the total sum of squares.

To evaluate the relation between quality parameters (PC1 and PC2 scores) and C and N losses from intact litter and faeces separately, we determined the relations between intact litter and faeces C and N losses and their scores on PC1 and PC2 with simple linear regressions and visualised these relations by fitting these variables as supplementary variables on the PCA.

For all statistical tests on C and N losses, block was included in the model as a random variable. All data were checked for normal distribution and homoscedasticity of residuals. All analyses were performed using the R software (version 3.5.3).

### Reporting summary

Further information on research design is available in the Nature Research Reporting Summary linked to this article.

## Supplementary information

Supplementary Information

Reporting Summary

## Data Availability

The data sets generated in this study are available from the University of Stirling’s online data repository (http://hdl.handle.net/11667/161).

## Code availability

The codes used to analyse the data sets of this study are available from the corresponding author upon reasonable request.
