# Supplementary material for: Detritivore conversion of litter into faeces accelerates organic matter turnover
Source: Commun Biol. 2020 Nov 11;3:660. doi: 10.1038/s42003-020-01392-4 (PMC7658975; doi:10.1038/s42003-020-01392-4)
Supplement: Supplementary file 1 — Supplementary Information [file 42003_2020_1392_MOESM1_ESM.pdf]

Supplementary table 1: Chemical and physical characteristics of all substrate types (6 intact litter and 36 faeces types, see Fig. 1) including (a) Carbon to Nitrogen ratio, (b) tannin concentration, (c) dissolved organic carbon and (d) total dissolved nitrogen concentrations, (e) specific area of leaves and faecal pellets, (f) specific area of leaves and faeces particles, (g) water-holding capacity, (h) Alkyl C content, (i) O:N Alkyl C content, (j) Aryl C content, (k) Carboxyl C content, (l) Alkyl C to O:N Alkyl C ratio. For (a) to (g), n = 3 except for averages across detritivore or litter species (n = 18), and for the average across all detritivore and litter species (n = 108). For (h) to (l), n = 1 for all subtrates (absolute value; measured on a composite sample combining all three replicates) except for average across detritivore or litter species (n = 6), and for the average across all detritivore and litter species (n = 36).

(a) Carbon to Nitrogen ratio

Mean ± standard error

	Litter species						
**Substrate type**	*Acer*	*Aesculus*	*Corylus*	*Fagus*	*Quercus*	*Tilia*	*All litter*
Glomeris faeces	**20.3** ± 0.07	**23.9** ± 0.07	**20.5** ± 0.14	**36.2** ± 0.54	**22.8** ± 0.25	**30.1** ± 0.50	**25.6** ± 1.40
Ommatoiulus faeces	**20.2** ± 0.24	**25.8** ± 0.11	**21.7** ± 0.39	**33.4** ± 0.03	**24.9** ± 0.19	**27.9** ± 0.44	**25.6** ± 1.05
Tachypodoiulus faeces	**20.2** ± 0.35	**23.2** ± 0.12	**21.3** ± 0.12	**28.5** ± 0.25	**23.9** ± 0.21	**27.1** ± 0.41	**24.0** ± 0.72
Armadillidium faeces	**18.2** ± 0.16	**25.9** ± 0.27	**19.7** ± 0.26	**29.2** ± 0.11	**21.3** ± 0.68	**29.0** ± 0.47	**23.9** ± 1.07
Porcellio faeces	**17.7** ± 0.11	**21.4** ± 0.17	**19.2** ± 0.10	**25.4** ± 0.14	**20.6** ± 0.19	**26.1** ± 0.30	**21.7** ± 0.75
Cepaea faeces	**19.8** ± 0.05	**20.5** ± 0.14	**19.7** ± 0.25	**27.5** ± 0.03	**24.1** ± 0.27	**23.6** ± 1.07	**22.5** ± 0.70
**All faeces**	**19.3** ± 0.26	**23.5** ± 0.50	**20.3** ± 0.23	**30.0** ± 0.89	**22.9** ± 0.39	**27.3** ± 0.55	**23.9** ± 0.42
**Intact litter**	**17.6** ± 0.07	**34.4** ± 1.15	**22.5** ± 0.27	**40.8** ± 0.07	**19.7** ± 0.94	**33.8** ± 0.44	**28.1** ± 2.10

(b) Tannin concentrations (mg/g)

Mean ± standard error

	Litter species						
**Substrate type**	*Acer*	*Aesculus*	*Corylus*	*Fagus*	*Quercus*	*Tilia*	*All litter*
Glomeris faeces	**3.78** ± 0.82	**6.72** ± 0.90	**5.40** ± 1.22	**7.89** ± 0.84	**4.18** ± 1.39	**4.26** ± 1.64	**5.37** ± 0.54
Ommatoiulus faeces	**4.36** ± 1.73	**6.18** ± 1.36	**4.95** ± 0.32	**4.39** ± 0.90	**5.06** ± 0.37	**3.92** ± 0.51	**4.81** ± 0.39
Tachypodoiulus faeces	**3.66** ± 1.46	**3.94** ± 0.12	**3.74** ± 0.53	**3.93** ± 0.31	**2.32** ± 0.52	**3.26** ± 0.98	**3.48** ± 0.30
Armadillidium faeces	**3.81** ± 1.35	**10.46** ± 1.03	**4.23** ± 1.47	**4.61** ± 0.48	**3.06** ± 1.06	**5.88** ± 2.15	**5.34** ± 0.75
Porcellio faeces	**5.44** ± 1.10	**8.97** ± 2.10	**3.81** ± 0.59	**3.46** ± 0.49	**3.17** ± 0.78	**2.14** ± 1.18	**4.50** ± 0.67
Cepaea faeces	**5.84** ± 0.30	**5.13** ± 0.91	**4.41** ± 0.08	**4.03** ± 0.18	**4.58** ± 0.35	**3.12** ± 0.89	**4.52** ± 0.28
**All faeces**	**4.33** ± 0.47	**6.90** ± 0.68	**4.42** ± 0.33	**4.72** ± 0.41	**3.73** ± 0.37	**3.76** ± 0.54	**4.67** ± 0.22
**Intact litter**	**9.21** ± 1.44	**24.73** ± 2.63	**3.57** ± 0.13	**12.18** ± 0.84	**5.23** ± 1.44	**3.58** ± 0.73	**9.75** ± 1.86

(c) Dissolved organic carbon content (mg/g)

Mean ± standard error

Substrate type	Litter species						
	Acer	*Aesculus*	*Corylus*	*Fagus*	*Quercus*	*Tilia*	*All litter*
Glomeris faeces	**19.16** ± 0.79	**4.14** ± 0.23	**6.28** ± 0.15	**6.33** ± 0.09	**13.8** ± 0.53	**4.38** ± 0.26	**9.02** ± 1.36
Ommatoiulus faeces	**15.50** ± 0.18	**4.28** ± 0.20	**5.92** ± 0.28	**9.31** ± 0.19	**12.71** ± 0.34	**4.21** ± 0.17	**8.66** ± 1.04
Tachypodoiulus faeces	**14.79** ± 0.11	**4.40** ± 0.22	**6.30** ± 0.11	**8.57** ± 0.18	**11.58** ± 0.21	**4.07** ± 0.16	**8.28** ± 0.94
Armadillidium faeces	**21.15** ± 0.70	**5.17** ± 0.33	**6.46** ± 0.22	**9.64** ± 0.41	**13.48** ± 0.13	**5.02** ± 0.06	**10.15** ± 1.40
Porcellio faeces	**26.44** ± 0.66	**8.22** ± 0.05	**8.78** ± 0.13	**13.58** ± 0.56	**17.72** ± 0.66	**6.67** ± 0.20	**13.57** ± 1.67
Cepaea faeces	**18.86** ± 1.99	**5.04** ± 0.56	**5.16** ± 0.11	**4.39** ± 0.55	**8.51** ± 0.06	**4.27** ± 0.54	**7.7** ± 1.30
**All faeces**	**19.40** ± 0.99	**5.21** ± 0.36	**6.48** ± 0.28	**8.64** ± 0.71	**12.96** ± 0.68	**4.77** ± 0.24	**9.56** ± 0.55
**Intact litter**	**13.93** ± 0.60	**3.51** ± 0.18	**7.61** ± 0.37	**5.30** ± 0.11	**7.24** ± 0.23	**5.19** ± 0.20	**7.13** ± 0.82

(d) Total dissolved nitrogen content (mg/g)

Mean ± standard error

Substrate type	Litter species						
	Acer	*Aesculus*	*Corylus*	*Fagus*	*Quercus*	*Tilia*	*All litter*
Glomeris faeces	**3.60** ± 0.19	**0.97** ± 0.04	**1.49** ± 0.05	**1.25** ± 0.04	**2.70** ± 0.08	**0.94** ± 0.05	**1.83** ± 0.24
Ommatoiulus faeces	**2.99** ± 0.11	**0.68** ± 0.07	**1.40** ± 0.05	**0.94** ± 0.02	**1.68** ± 0.09	**0.54** ± 0.04	**1.37** ± 0.20
Tachypodoiulus faeces	**2.64** ± 0.08	**0.68** ± 0.10	**1.14** ± 0.05	**1.21** ± 0.05	**1.57** ± 0.10	**0.57** ± 0.06	**1.30** ± 0.17
Armadillidium faeces	**4.54** ± 0.05	**1.90** ± 0.13	**2.14** ± 0.09	**1.91** ± 0.09	**3.14** ± 0.04	**1.53** ± 0.07	**2.53** ± 0.25
Porcellio faeces	**4.89** ± 0.25	**2.39** ± 0.09	**2.55** ± 0.03	**2.26** ± 0.12	**3.84** ± 0.21	**1.82** ± 0.11	**2.96** ± 0.26
Cepaea faeces	**2.33** ± 0.28	**0.99** ± 0.07	**1.60** ± 0.18	**0.77** ± 0.04	**1.51** ± 0.12	**0.86** ± 0.08	**1.34** ± 0.14
**All faeces**	**3.55** ± 0.24	**1.27** ± 0.16	**1.72** ± 0.12	**1.39** ± 0.13	**2.41** ± 0.22	**1.05** ± 0.12	**1.89** ± 0.11
**Intact litter**	**3.39** ± 0.05	**0.78** ± 0.07	**1.37** ± 0.06	**0.69** ± 0.06	**1.01** ± 0.05	**0.55** ± 0.09	**1.30** ± 0.24

(e) Specific area of leaves and faecal pellets (mm^2/mg)

Mean ± standard error

Substrate type	Litter species: *Acer*	*Aesculus*	*Corylus*	*Fagus*	*Quercus*	*Tilia*	*All litter*
Glomeris faeces	**4.80** ± 0.26	**5.05** ± 0.38	**4.08** ± 0.16	**5.67** ± 0.66	**5.57** ± 0.20	**3.67** ± 0.16	**4.81** ± 0.21
Ommatoiulus faeces	**4.59** ± 0.17	**4.82** ± 0.08	**4.58** ± 0.10	**6.39** ± 0.19	**5.61** ± 0.13	**4.28** ± 0.06	**5.04** ± 0.18
Tachypodoiulus faeces	**4.61** ± 0.05	**5.67** ± 0.1	**5.11** ± 0.08	**6.40** ± 0.19	**5.63** ± 0.07	**5.08** ± 0.17	**5.42** ± 0.14
Armadillidium faeces	**5.54** ± 0.11	**5.20** ± 0.15	**5.16** ± 0.01	**6.49** ± 0.15	**6.00** ± 0.22	**4.92** ± 0.09	**5.55** ± 0.14
Porcellio faeces	**6.84** ± 0.06	**7.27** ± 0.08	**7.05** ± 0.02	**8.50** ± 0.17	**7.92** ± 0.13	**6.96** ± 0.03	**7.42** ± 0.15
Cepaea faeces	**3.90** ± 0.31	**3.93** ± 0.22	**3.49** ± 0.2	**4.10** ± 0.27	**4.55** ± 0.39	**2.65** ± 0.08	**3.77** ± 0.17
**All faeces**	**5.06** ± 0.24	**5.32** ± 0.26	**4.91** ± 0.27	**6.26** ± 0.33	**5.88** ± 0.26	**4.59** ± 0.33	**5.34** ± 0.13
**Intact litter**	**14.78** ± 0.86	**8.70** ± 1.97	**11.83** ± 0.84	**23.55** ± 2.97	**17.81** ± 1.48	**11.62** ± 0.22	**14.72** ± 1.31

(f) Specific area of leaves and faeces particles (mm^2/mg)

Mean ± standard error

Substrate type	Litter species: *Acer*	*Aesculus*	*Corylus*	*Fagus*	*Quercus*	*Tilia*	*All litter*
Glomeris faeces	**34.86** ± 0.77	**46.20** ± 1.69	**28.30** ± 3.78	**58.43** ± 3.86	**42.42** ± 3.56	**31.61** ± 3.36	**40.30** ± 2.68
Ommatoiulus faeces	**38.48** ± 1.90	**39.10** ± 3.20	**34.21** ± 0.99	**58.04** ± 4.03	**53.31** ± 8.69	**34.70** ± 2.17	**42.97** ± 2.69
Tachypodoiulus faeces	**44.77** ± 4.29	**57.40** ± 2.58	**45.05** ± 7.58	**59.83** ± 3.3	**44.20** ± 2.57	**42.21** ± 2.32	**48.91** ± 2.22
Armadillidium faeces	**40.83** ± 5.62	**65.27** ± 8.64	**42.85** ± 3.21	**65.78** ± 4.75	**46.24** ± 1.74	**44.43** ± 2.65	**50.90** ± 3.06
Porcellio faeces	**70.84** ± 4.58	**75.39** ± 7.68	**56.65** ± 3.67	**47.52** ± 1.35	**47.07** ± 2.51	**41.03** ± 0.86	**56.42** ± 3.40
Cepaea faeces	**37.96** ± 2.06	**27.67** ± 2.73	**43.24** ± 2.48	**47.47** ± 0.86	**33.61** ± 1.34	**34.15** ± 9.43	**37.35** ± 2.16
**All faeces**	**44.41** ± 3.19	**51.84** ± 4.28	**41.72** ± 2.58	**56.18** ± 1.98	**44.48** ± 2.03	**38.02** ± 1.91	**46.14** ± 1.26
**Intact litter**	**14.78** ± 0.86	**8.70** ± 1.97	**11.83** ± 0.84	**23.55** ± 2.97	**17.81** ± 1.48	**11.62** ± 0.22	**14.72** ± 1.31

(g) Water-holding capacity (g H_2O/g)

Mean ± standard error

Substrate type	Litter species: *Acer*	*Aesculus*	*Corylus*	*Fagus*	*Quercus*	*Tilia*	*All litter*
Glomeris faeces	**2.78** ± 0.18	**2.61** ± 0.11	**2.59** ± 0.12	**2.58** ± 0.21	**2.94** ± 0.10	**2.38** ± 0.08	**2.65** ± 0.06
Ommatoiulus faeces	**2.74** ± 0.11	**2.28** ± 0.09	**2.56** ± 0.11	**3.22** ± 0.06	**2.70** ± 0.04	**2.30** ± 0.10	**2.63** ± 0.08
Tachypodoiulus faeces	**2.08** ± 0.10	**2.49** ± 0.03	**2.18** ± 0.08	**2.68** ± 0.06	**2.35** ± 0.08	**2.10** ± 0.02	**2.31** ± 0.06
Armadillidium faeces	**2.59** ± 0.03	**2.16** ± 0.08	**2.07** ± 0.21	**2.69** ± 0.13	**2.68** ± 0.07	**2.30** ± 0.05	**2.42** ± 0.07
Porcellio faeces	**2.13** ± 0.08	**1.79** ± 0.16	**2.07** ± 0.07	**3.18** ± 0.06	**2.80** ± 0.03	**2.06** ± 0.02	**2.34** ± 0.12
Cepaea faeces	**2.68** ± 0.15	**2.73** ± 0.15	**2.82** ± 0.18	**2.57** ± 0.06	**2.21** ± 0.08	**2.73** ± 0.11	**2.62** ± 0.07
All faeces	**2.51** ± 0.08	**2.35** ± 0.09	**2.38** ± 0.08	**2.82** ± 0.08	**2.61** ± 0.07	**2.31** ± 0.06	**2.50** ± 0.03
Intact litter	**3.26** ± 0.09	**1.53** ± 0.09	**2.57** ± 0.23	**3.21** ± 0.14	**2.68** ± 0.22	**2.75** ± 0.06	**2.67** ± 0.15

(h) Alkyl C (%)

Exact value for substrates; mean ± standard error for averages across species of detritivores and/or litter

Substrate type	Litter species: *Acer*	*Aesculus*	*Corylus*	*Fagus*	*Quercus*	*Tilia*	*All litter*
Glomeris faeces	**19.90**	**16.20**	**16.20**	**9.00**	**16.30**	**16.30**	**15.65** ± 1.46
Ommatoiulus faeces	**21.90**	**16.10**	**16.50**	**12.80**	**17.70**	**17.60**	**17.10** ± 1.20
Tachypodoiulus faeces	**19.90**	**17.20**	**16.60**	**17.40**	**17.30**	**16.30**	**17.45** ± 0.52
Armadillidium faeces	**19.60**	**14.00**	**18.00**	**15.60**	**12.90**	**16.60**	**16.12** ± 1.02
Porcellio faeces	**19.20**	**17.60**	**16.80**	**16.70**	**16.60**	**15.20**	**17.02** ± 0.54
Cepaea faeces	**20.10**	**8.60**	**16.40**	**16.50**	**17.40**	**16.50**	**15.92** ± 1.57
All faeces	**20.10** ± 0.38	**14.95** ± 1.37	**16.75** ± 0.26	**14.67** ± 1.31	**16.37** ± 0.73	**16.42** ± 0.31	**16.54** ± 0.44
Intact litter	**18.50**	**12.80**	**17.40**	**12.40**	**15.50**	**13.80**	**15.07** ± 1.02

(i) O:N Alkyl C (%)

Exact value for substrates; mean ± standard error for averages across species of detritivores and/or litter

Substrate type	Litter species: *Acer*	*Aesculus*	*Corylus*	*Fagus*	*Quercus*	*Tilia*	*All litter*
Glomeris faeces	54.70	48.60	56.10	57.20	56.50	54.50	54.60 ± 1.27
Ommatoiulus faeces	55.30	48.70	56.00	65.80	57.10	54.70	56.27 ± 2.25
Tachypodoiulus faeces	53.80	51.60	55.60	54.30	47.40	54.30	52.83 ± 1.21
Armadillidium faeces	53.50	49.00	58.80	68.10	61.60	57.90	58.15 ± 2.69
Porcellio faeces	57.30	50.60	56.90	56.40	53.00	53.60	54.63 ± 1.09
Cepaea faeces	54.00	47.90	58.20	43.80	57.80	58.50	53.37 ± 2.52
All faeces	54.77 ± 0.57	49.40 ± 0.57	56.93 ± 0.53	57.60 ± 3.56	55.57 ± 1.98	55.58 ± 0.84	54.98 ± 0.80
Intact litter	56.10	48.40	56.00	61.00	56.90	55.40	55.63 ± 1.66

(j) Aryl C (%)

Exact value for substrates; mean ± standard error for averages across species of detritivores and/or litter

Substrate type	Litter species: *Acer*	*Aesculus*	*Corylus*	*Fagus*	*Quercus*	*Tilia*	*All litter*
Glomeris faeces	16.60	27.10	19.30	22.70	18.00	22.70	21.07 ± 1.57
Ommatoiulus faeces	15.50	27.50	19.20	16.50	17.40	21.50	19.60 ± 1.80
Tachypodoiulus faeces	17.20	24.60	19.00	18.40	21.70	21.30	20.37 ± 1.10
Armadillidium faeces	18.10	28.60	17.70	14.20	19.00	21.30	19.82 ± 1.99
Porcellio faeces	15.90	24.40	17.90	16.60	19.60	22.60	19.50 ± 1.39
Cepaea faeces	17.40	28.40	17.30	22.50	17.70	19.60	20.48 ± 1.78
All faeces	16.78 ± 0.40	26.77 ± 0.75	18.40 ± 0.35	18.48 ± 1.41	18.90 ± 0.65	21.50 ± 0.46	20.14 ± 0.62
Intact litter	18.50	33.20	19.60	21.40	19.30	24.20	22.70 ± 2.26

(k) Carboxyl C (%)

Exact value for substrates; mean ± standard error for averages across species of detritivores and/or litter

	Litter species						
**Substrate type**	***Acer***	***Aesculus***	***Corylus***	***Fagus***	***Quercus***	***Tilia***	***All litter***
Glomeris faeces	8.36	7.54	8.27	10.65	8.99	5.95	8.29 ± 0.64
Ommatoiulus faeces	7.45	7.33	8.15	4.69	7.15	6.26	6.84 ± 0.50
Tachypodoiulus faeces	8.94	6.78	8.42	9.79	13.01	7.88	9.14 ± 0.88
Armadillidium faeces	8.98	7.92	5.40	2.00	5.97	4.30	5.76 ± 1.02
Porcellio faeces	7.12	7.18	8.24	9.59	10.39	8.33	8.48 ± 0.53
Cepaea faeces	8.59	13.93	7.91	16.30	6.60	5.03	9.73 ± 1.80
**All faeces**	8.24 ± 0.32	8.45 ± 1.11	8.12 ± 0.47	8.68 ± 2.04	9.10 ± 1.09	6.69 ± 0.64	8.04 ± 0.44
**Intact litter**	7.15	5.08	6.78	5.23	8.16	6.43	6.47 ± 0.48

(f) Alkyl C to O:N Alkyl C ratio

Exact value for substrates; mean ± standard error for averages across species of detritivores and/or litter

	Litter species						
**Substrate type**	***Acer***	***Aesculus***	***Corylus***	***Fagus***	***Quercus***	***Tilia***	***All litter***
Glomeris faeces	0.36	0.33	0.29	0.16	0.29	0.30	0.29 ± 0.03
Ommatoiulus faeces	0.40	0.33	0.29	0.19	0.31	0.32	0.31 ± 0.03
Tachypodoiulus faeces	0.37	0.33	0.30	0.32	0.37	0.30	0.33 ± 0.01
Armadillidium faeces	0.37	0.29	0.31	0.23	0.21	0.29	0.28 ± 0.02
Porcellio faeces	0.34	0.35	0.29	0.30	0.31	0.28	0.31 ± 0.01
Cepaea faeces	0.37	0.18	0.28	0.38	0.30	0.28	0.30 ± 0.03
**All faeces**	0.37 ± 0.01	0.30 ± 0.03	0.29 ± 0.01	0.26 ± 0.03	0.30 ± 0.02	0.30 ± 0.01	0.30 ± 0.01
**Intact litter**	0.33	0.26	0.31	0.20	0.27	0.25	0.27 ± 0.02
