# Supplementary material for: Detritivore conversion of litter into faeces accelerates organic matter turnover
Source: Commun Biol. 2020 Nov 11;3:660. doi: 10.1038/s42003-020-01392-4 (PMC7658975; doi:10.1038/s42003-020-01392-4)
Supplement: Supplementary file 2 — Reporting Summary [file 42003_2020_1392_MOESM2_ESM.pdf]

nature research

Corresponding author(s): François-Xavier Joly

Last updated by author(s): Jun 5, 2020

Reporting Summary

Nature Research wishes to improve the reproducibility of the work that we publish. This form provides structure for consistency and transparency in reporting. For further information on Nature Research policies, see our Editorial Policies and the Editorial Policy Checklist.

Statistics

For all statistical analyses, confirm that the following items are present in the figure legend, table legend, main text, or Methods section.

n/a	Confirmed	
[ ]	[x]	The exact sample size (*n*) for each experimental group/condition, given as a discrete number and unit of measurement
[ ]	[x]	A statement on whether measurements were taken from distinct samples or whether the same sample was measured repeatedly
[ ]	[x]	The statistical test(s) used AND whether they are one- or two-sided<br>*Only common tests should be described solely by name; describe more complex techniques in the Methods section.*
[x]	[ ]	A description of all covariates tested
[ ]	[x]	A description of any assumptions or corrections, such as tests of normality and adjustment for multiple comparisons
[ ]	[x]	A full description of the statistical parameters including central tendency (e.g. means) or other basic estimates (e.g. regression coefficient) AND variation (e.g. standard deviation) or associated estimates of uncertainty (e.g. confidence intervals)
[ ]	[x]	For null hypothesis testing, the test statistic (e.g. *F*, *t*, *r*) with confidence intervals, effect sizes, degrees of freedom and *P* value noted<br>*Give P values as exact values whenever suitable.*
[x]	[ ]	For Bayesian analysis, information on the choice of priors and Markov chain Monte Carlo settings
[x]	[ ]	For hierarchical and complex designs, identification of the appropriate level for tests and full reporting of outcomes
[ ]	[x]	Estimates of effect sizes (e.g. Cohen's *d*, Pearson's *r*), indicating how they were calculated

Our web collection on statistics for biologists contains articles on many of the points above.

Software and code

Policy information about availability of computer code

Data collection	ImageJ, version 1.46r
Data analysis	R software (version 3.5.3).

For manuscripts utilizing custom algorithms or software that are central to the research but not yet described in published literature, software must be made available to editors and reviewers. We strongly encourage code deposition in a community repository (e.g. GitHub). See the Nature Research guidelines for submitting code & software for further information.

Data

Policy information about availability of data

All manuscripts must include a data availability statement. This statement should provide the following information, where applicable:

- Accession codes, unique identifiers, or web links for publicly available datasets
- A list of figures that have associated raw data
- A description of any restrictions on data availability

The data that support the findings of this study are available from the corresponding author upon request.

Field-specific reporting

Please select the one below that is the best fit for your research. If you are not sure, read the appropriate sections before making your selection.

☐ Life sciences ☐ Behavioural & social sciences ☒ Ecological, evolutionary & environmental sciences

For a reference copy of the document with all sections, see nature.com/documents/nr-reporting-summary-flat.pdf

Ecological, evolutionary & environmental sciences study design

All studies must disclose on these points even when the disclosure is negative.

Study description	We fed leaf litter from six tree species to six animal species, separately, and compared the quality and decomposition rates of the resulting 36 faeces types and 6 intact litter type as control. Physico-chemical analyses for all 42 substrates types were performed on three replicates (108 samples). Decomposition rates for all 42 substrate types were performed on five replicates (210 samples).
Research sample	Soil animals (Glomeris marginata, Ommatoiulus sabulosus, Tachypodoiulus niger, Porcellio scaber, Armadillidium vulgare, Cepaea nemoralis) and plant species (Acer pseudoplatanus, Aesculus hippocastanum, Corylus avellana, Fagus sylvatica, Quercus robur, Tilia Platyphyllos) were collected in various sites across the Scottish Lowlands. They were chosen as they are widely distributed species accross Europe, occurring in temperate and Mediterranean ecosystems, and represent large phylogenetic gradient (for animal and plant species) and a large litter quality gradient (for plant species).
Sampling strategy	We chose six plant species and six animal species as six points along a continous axis give much greater power in regression designs than five points or less. For physicochemical measures (that were repeatable in case of failed measurement), analyses were done on three replicates per treatment in order to provide sufficient sample size for statistical analyses. For the decomposition incubation, five replicates per treatment were set up, in order to have sufficient sample size for statistical analyses even in case of accidental loss of one or two replicates. However, this was not the case. This sample size choice was based on a successful previous experiment (Joly et al. 2018 Functional Ecology).
Data collection	François-Xavier Joly collected the litter and animals, fed all litter species to all animal species separately and collected the 36 36 resulting faeces and 6 intact litter controls. On the 42 substrate types:<br>-François-Xavier Joly and Mathieu Coulis took high-resolution photographs and measured the litter and faeces specific area.<br>-François-Xavier Joly and Sylvain Coq measured the tannin concentrations.<br>-François-Xavier Joly, Isabel Prater and Carsten W. Mueller measured the 13C-NMR spectra.<br>-François-Xavier Joly measured the dissolved organic carbon and nitrogen concentration, water-holding capacity and fragmentation levels<br>-François-Xavier Joly measured the decomposition rates by incubating substrates in microcosms for 180 days, collecting the remaining decomposed litter and faeces, grinding them and analysing them for C and N.
Timing and spatial scale	Litter samples and animals were collected in May and June 2018 which corresponds to a period of peak activity for these animals. All litter was fed to all animals separately over a one-month period to allow animals, for each plant-animal combination, to produce sufficient faeces for further analyses while limiting changes in litter quality due to microbial decomposition. All substrate types were incubated over 180 days at 22°C and 70% soil water holding capacity to ensure similar level of mass loss with previous studies.
Data exclusions	No data was excluded.
Reproducibility	For each measure, all samples were analysed alongside standards for measure quality control. The low variability in measure values amongst replicates of a given treatment gives high confidence in the reproducibility of the measures.
Randomization	Treatments were randomly attributed to microscosms which were organised by block according to a complete randomised block design.
Blinding	After the faeces collection process during which blinding was not possible (handler could identify plant and animal species), all samples were attributed a code which was used for sample handling during the incubation and measurements.

Did the study involve field work? ☒ Yes ☐ No

Field work, collection and transport

Field conditions	Fieldwork was carried out over several days in May-June 2018, with temperature ranging 14-20°C and no rainfall.
Location	Peebles, UK (55°38'45.8"N, 3°07'55.4"W); Dunfermline, UK (56°02'23.7"N, 3°19'49.2"W); Dundee, UK (56°32'08.5"N, 3°01'51.9"W); Dunfermline, UK (56°01'35.3"N 3°23'14.1"W); Stirling, UK (56°07'26.7"N, 3°55'51.2"W); Stirling, UK (56°08'07.3"N, 3°55'16.3"W); Stirling, UK (56°08'29.5"N, 3°55'14.2"W)
Access & import/export	All sites were freely accessible.
Disturbance	No disturbance was caused by the sample collection.

Reporting for specific materials, systems and methods

We require information from authors about some types of materials, experimental systems and methods used in many studies. Here, indicate whether each material, system or method listed is relevant to your study. If you are not sure if a list item applies to your research, read the appropriate section before selecting a response.

Materials & experimental systems

n/a	Involved in the study
☒	☐ Antibodies
☒	☐ Eukaryotic cell lines
☒	☐ Palaeontology and archaeology
☐	☒ Animals and other organisms
☒	☐ Human research participants
☒	☐ Clinical data
☒	☐ Dual use research of concern

Methods

n/a	Involved in the study
☒	☐ ChIP-seq
☒	☐ Flow cytometry
☒	☐ MRI-based neuroimaging

Animals and other organisms

Policy information about studies involving animals; ARRIVE guidelines recommended for reporting animal research

Laboratory animals	No labobatory animals were involved.
Wild animals	Millipedes (Glomeris marginata, Ommatoiulus sabulosus, Tachypodoiulus niger), isopods (Porcellio scaber, Armadillidium vulgare) and snails (Cepaea nemoralis) were collected by hand. They were transported to the laboratory in plastic containers containing leaf litter from the site of origin. Animals were released back at their site of origin after the experiment.
Field-collected samples	Leaf litter samples were transferred to the laboratory in cardboard boxes. Litter was air-dried and used for the incubation experiment.
Ethics oversight	No ethical approval or guidance was required, as the UK animal act of 1986 applies to vertebrates, leaving good practice on invertebrate research at the discretion of the researcher.

Note that full information on the approval of the study protocol must also be provided in the manuscript.
